# Uncoupling Proteins as Therapeutic Targets for Neurodegenerative Diseases

**DOI:** 10.3390/ijms23105672

**Published:** 2022-05-18

**Authors:** Colin J. Barnstable, Mingliang Zhang, Joyce Tombran-Tink

**Affiliations:** 1 Department of Neural and Behavioral Sciences, Penn State College of Medicine, 500 University Drive, Hershey, PA 17033, USA; jttink@aol.com; 2 Tianjin Key Laboratory of Retinal Functions and Diseases, Tianjin Branch of National Clinical Research Center for Ocular Disease, Eye Institute and School of Optometry, Tianjin Medical University Eye Hospital, 251 Fukang Road, Tianjin 300384, China; zmlpku@163.com

**Keywords:** oxidative stress, reactive oxygen species (ROS), uncoupling protein 2 (UCP2)

## Abstract

Most of the major retinal degenerative diseases are associated with significant levels of oxidative stress. One of the major sources contributing to the overall level of stress is the reactive oxygen species (ROS) generated by mitochondria. The driving force for ROS production is the proton gradient across the inner mitochondrial membrane. This gradient can be modulated by members of the uncoupling protein family, particularly the widely expressed UCP2. The overexpression and knockout studies of UCP2 in mice have established the ability of this protein to provide neuroprotection in a number of animal models of neurological disease, including retinal diseases. The expression and activity of UCP2 are controlled at the transcriptional, translational and post-translational levels, making it an ideal candidate for therapeutic intervention. In addition to regulation by a number of growth factors, including the neuroprotective factors LIF and PEDF, small molecule activators of UCP2 have been found to reduce mitochondrial ROS production and protect against cell death both in culture and animal models of retinal degeneration. Such studies point to the development of new therapeutics to combat a range of blinding retinal degenerative diseases and possibly other diseases in which oxidative stress plays a key role.

## 1. Introduction

Mitochondria serve not only as the energy converters of a cell but also as sensitive monitors of health and stress. Metabolites passing through the mitochondria provide a continuous readout of any stress or abnormal conditions. Numerous homeostatic mechanisms can moderate the effects of stresses, although in extreme cases, these can be overwhelmed and lead to a mitochondrial initiated apoptotic cascade and cell death. One of the major stressors that affect mitochondria is oxidative stress, a condition accompanying many neurological diseases or injury. In this review we define oxidative stress as an imbalance between the production and detoxification of reactive oxygen species (ROS). ROS are both the results of external stress and internal metabolism, since normal mitochondrial function also generates ROS as an obligatory part of ATP production. The major source of ROS production by the electron transport chain is Complex I, although Complex III can also produce ROS (see Refs [1,2,3] for a more detailed discussion). Although not discussed in this review, mitochondria can also be damaged by excess production of the reactive nitrogen species NO and ONOO-[4,5]. The electron transport chain can catalyze the reduction of nitrite to NO, and while NO is usually protective, excess amounts can lead to damage of mitochondria. This makes these organelles continuously at risk of the damaging consequences of oxidative stress.

Among the most extensively studied homeostatic regulators of mitochondrial stress are the uncoupling proteins. Originally studied in detail because of the role of the prototypic member of this family, UCP1, in thermogenesis in rodent brown fat, it is now clear that this family plays an important role in many tissues and has great potential as a therapeutic target [1,2,3]. The UCP family has been defined in many species and mammals as consisting of five homologous proteins, UCP1 to UCP5 (an additional member, UCP6, has been found in invertebrates). More generally, UCPs belong to the SLC25 superfamily of mitochondrial solute carrier genes, which has 53 members, all encoded in nuclear DNA. As discussed below, these proteins may be multifunctional and carry out essential functions in both regulating ROS production and substrate availability.

Understanding the relationships among members of the UCPs, their role in normal cellular metabolism and their response to stress and disease will be an advantage to elucidate ways in which oxidative stress can exacerbate, or even initiate, disease pathology and potentially lead to new approaches to combat it. In this review, we summarize a number of studies investigating UCP function in several animal neurological disease models, as well as our early studies identifying compounds that can alleviate neuronal degeneration by activating UCPs. Because of the wealth of data showing the role of UCP2 in neurological disease and neuroprotection, this review is focused on this member of the uncoupling protein family. In addition, until recently, the primary way of manipulating uncoupling proteins has been by overexpression or knockout studies. This has resulted in most studies being carried out in mice, although we are well aware that there are species differences in UCP expression patterns, and possibly in UCP function, between species. Although UCP2 was first described in 1997 [6], we focused on more recent experimental studies, using references to review articles that can provide a fuller historical perspective.

This article is not intended to be a comprehensive review of uncoupling proteins. In a review of 1101 published studies, evidence in favor of UCP2 and UCP3 reducing oxidative stress was found, but a number of studies also provided evidence for UCP2/3 serving as Ca^2+^ transporters or as transporters of C4 metabolites, leaving open the mechanisms by which these proteins function (1). Other recent reviews have shown that UCP2 and UCP3 reduce ROS production in cardiovascular issue, and dysregulation of UCP2 can lead to cardiovascular disease [7,8]. UCP2 has also been linked to obesity, diabetes and cancer, though the genetic studies recently reviewed show an association but not a clear mechanism [9]. Although many mechanistic details still remain to be worked out, the overwhelming consensus is that activation of UCP2, and other uncoupling proteins, reduces oxidative stress and is beneficial for tissues. As discussed below, this provides a strong impetus for identifying therapeutic agents that can modulate uncoupling protein activity.

In this review, we only briefly touched on the bioenergetics of uncoupling protein activity. Two recent reviews cover this in far more depth and show how, in spite of the low concentrations of UCP2 in many tissues, its activation can produce substantial effects on ROS production [2,10]. Similarly, several recent articles point out the unique aspects of the nervous system that make regulation of ROS production critical [11,12,13]. These aspects include the high metabolic rate of neural tissue, reliance on glucose as an energy source and reduced regenerative capacity. As in other tissues, there is general agreement that activation of uncoupling proteins can reduce oxidative stress and protect neural cells. Although the focus of this review is on chronic neurodegenerative diseases, there is also evidence for a role of uncoupling proteins in acute brain injury [12].

UCP4 and UCP5 are expressed in the brain, but their function has yet to be fully defined. Recent reviews of uncoupling proteins in the brain have discussed these proteins but have not reached a clear conclusion as to their primary function [11,12,13]. As stated above, this article focuses on UCP2 and its role in neurodegenerative disease, with a focus on neurodegenerative diseases of the eye. It is also among the first to review the evidence that small molecule activators of UCP2 can prevent neuronal death in an animal model of retinal disease.

## 2. The Uncoupling Protein Family

**Figure 1:**
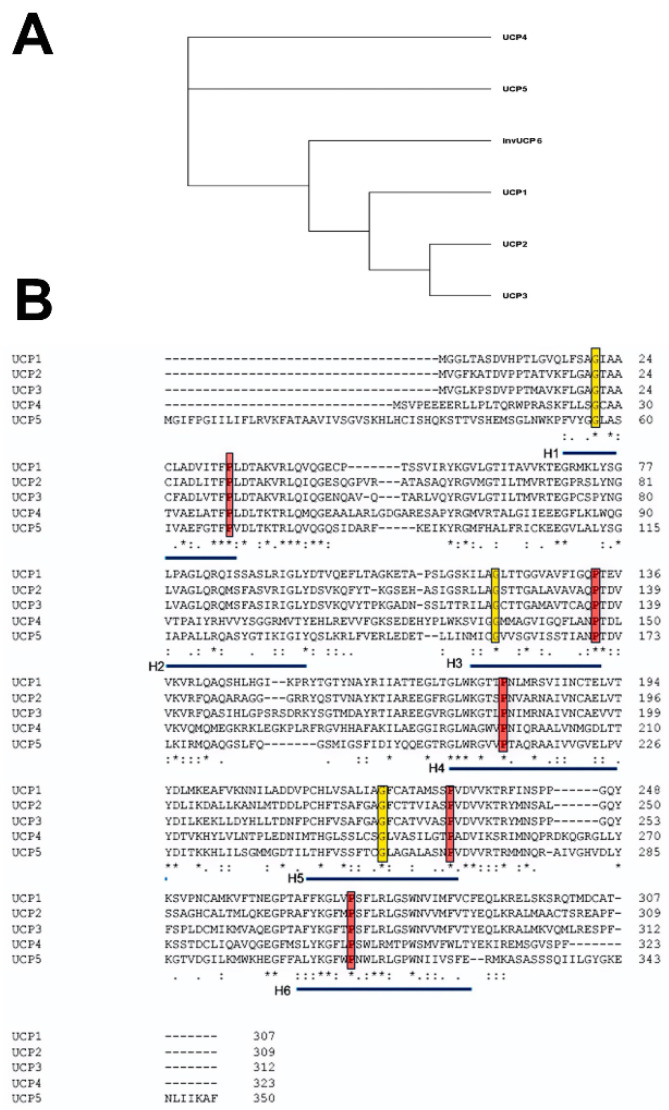
(**A**) A simplified phylogenetic tree of human UCPs. From the sequences in B, we derived a tree indicating the early divergence of UCP4 and 5 and a later divergence into invertebrate UCPs and a precursor of UCP1, 2 and 3. (**B**) Sequence alignment of the five human UCP proteins. Sequences used for alignment were the RefSeqs UCP1 (NP_068605.1), UCP2 (NP_003346.2), UCP3 (NP_003347.1), UCP4 (NP_004268.3) and UCP5 (XP_016885426.1). Alignment was carried out using the Clustal Omega program [26]. Conserved residues are marked with “*”, and conserved residues with “.” or “:”. The transmembrane helices are underlined in blue, key prolines in these helices outlined in red, and highly conserved glycines in yellow. Adapted from [2].

The five mammalian UCP members of the larger SLC25 gene family are numbered according to their order of discovery rather than closeness of relationship. Phylogenetic studies identified UCP paralogs in vertebrates, invertebrates and plants [13,14,15]. Based on nucleotide sequences, it has been proposed that UCP4 is closest to the ancestral gene, which diverged into three branches [16,17]. One branch gave rise to UCP4, one to UCP5 and a third to UCP1, UCP2 and UCP3 (Figure 1A). UCP2 and UCP3 genes show the closest homology and are adjacent to each other in the genome on human chromosome 11q13.4, suggesting a recent gene duplication event. An ongoing debate is whether the UCP family of proteins has a common set of functions or whether the evolutionary divergence is matched by a divergence of function.

**Figure 2:**
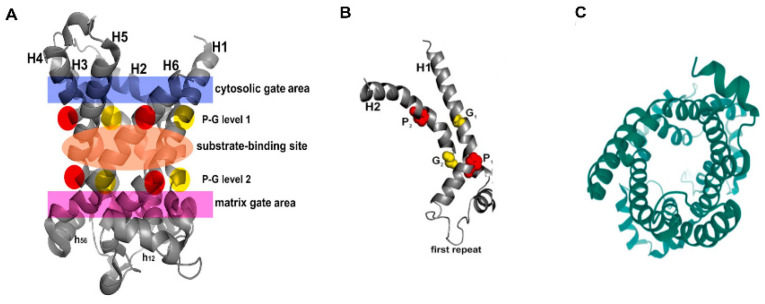
**Molecular models of uncoupling proteins.** (**A**) Crystal structure of the adenine nucleotide transporter from which most of the structural models of UCPs are derived. The 3D crystal structure of the carboxyatractyloside-ADP/ATP carrier complex (devoid of the inhibitor) is shown with the following regions in color: cytosolic gate (blue); substrate-binding site (orange); and matrix gate (purple). In P1–G1 and P2–G2, prolines are shown in red and glycines in yellow. (**B**) The crystal structure of the first repeat of UCP2. Key prolines are marked in red and glycines in yellow. (**C**) When rotated 90° and viewed at end on, UCP2 and other members of the SLC25 transporter family have a clear central pore. Adapted from Reference [2].

Alignment of the amino acid sequences of the five mammalian uncoupling proteins clearly indicates their homology and identifies a number of invariant residues (Figure 1B). For example, UCP2 encodes a protein of 309 amino acids, and UCP3, a protein of 312 amino acids, with 71% homology between them [18]. While there is currently no crystal structure of any UCP, by fitting their amino acid sequences to the known crystal structure of the bovine and Saccharomyces cerevisiae adenine nucleotide translocator proteins (ANT1), it was possible to obtain high-resolution models of UCP1 and UCP2 [19] (Figure 2A). In spite of the fact that ANTs and UCPs have only a <20% sequence identity, they have a similar domain structure and, particularly, a high conservation of certain key amino acid residues [20]. Based on these, the core UCP structure is defined as three repeating units, each with a loop of amino acids passing through the membrane twice, resulting in six transmembrane helices (H1 to H6 in Figure 2A, H1 and H2 in Figure 2B). In the odd-numbered helices, a conserved proline residue introduces a kink in the helix (highlighted in red), and a conserved glycine allows movement of the polypeptide chain (highlighted in yellow). Conserved prolines are also found in helices 4 and 6, and these further alter the helical structure. When rotated it can be seen that these helices are arranged around a central pore, the dynamic opening and closing of which is thought to regulate transport through the molecule (Figure 2C). A number of studies have further refined these molecular models and identified a series of residues important for the transport/uncoupling function, as well as for regulation by other molecules [20,21]. The structural features of uncoupling proteins have recently been elegantly reviewed, including a detailed discussion of whether these proteins exist as monomers, dimers or tetramers [22]. These studies provide a picture of a very dynamic molecule whose movements suggest an active transport process rather than simple opening and closing of a pore.

The structural similarities between the UCPs and other members of the SLC25 transporter family also raise the possibility of functional similarities. This is particularly true of the ANT family. The basic function of ANTs is to facilitate the exchange of ADP and ATP across the inner mitochondrial membrane. Normally, this involves the import of ADP and export of the ATP generated by oxidative phosphorylation. These molecules can, however, import ATP generated by glycolysis where it can be hydrolyzed by the F1F0-ATPase complex with the pumping out of a proton into the intermembrane space [23]. There is good evidence that these proteins can uncouple mitochondria, and the structural similarity with UCPs suggests that they may share a number of regulatory mechanisms [24,25]. As with UCPS, it is not clear whether this uncoupling activity represents proton migration through a central pore of the protein or a carrier mechanism utilizing fatty acids or some other molecule.

## 3. UCPs as Multifunctional Proteins

There is widespread agreement that the primary function of UCP1 is to allow protons to leak across the inner mitochondrial membrane and thus uncouple mitochondrial respiration from ATP synthesis. There is also strong evidence that UCP2 can uncouple mitochondrial respiration, but evidence for an uncoupling function of the other UCPs is less clear. Much of the debate has centered around whether UCPs have other metabolic functions in addition to, or instead of, transport of H^+^ ions as their primary function. For example, when reconstituted in liposomes, UCP2 will catalyze transport of phosphate, malate and aspartate [27]. Loss of function of UCP2 did not result in a significant increase in ROS production. This observation may be difficult to interpret because loss of UCP2 increases mitophagy and may alter the number of mitochondria generating ROS [28]. Studies using UCP2 null cells to examine the potential role of this uncoupling protein in metabolite transport provide evidence that these cells display a response associated with a metabolic switch from fatty acid oxidation to glucose metabolism, but only when sufficient glucose was available [29]. The physiological importance of this has been questioned by the observation that UCP2 null cells show no change in aspartate efflux from mitochondria [30]. UCP2 null cells do, however, show high levels of resting ROS production, and UCP2 null mice show increased susceptibility to the excitotoxins NMDA and kainate [31]. Other studies have provided evidence that UCP2 can alleviate oxidative stress by transporting Ca^2+^ into the mitochondrial matrix or by transporting C4 metabolites (reviewed in Ref [1]). There is still considerable debate about the relative importance of direct uncoupling activity vs. metabolite transport in the function of UCP2 and other uncoupling proteins.

A “combined” hypothesis of UCP action suggests uncoupling (proton transport) represents the primordial function of these proteins and that other transport and metabolic functions evolved over time [32]. The relative balance of these functions may differ among the various UCPs, with UCP1 primarily involved in proton transport and UCP4 and 5 in metabolic regulation. UCP2 (and UCP3) may combine these functions.

In this review, we will discuss the role of UCPs in neurodegenerative diseases and injury with a focus on UCP2 because it has the most direct evidence for a role in these conditions, and we know more about its structure and activation. UCP3 expression is primarily restricted to skeletal muscle, and there is currently little direct evidence for its involvement in any neurological disease. While UCP4 and UCP5 are expressed in the nervous system, there is currently less evidence that their activation can serve a neuroprotective role. While many of the genetic studies described below clearly indicate that UCP2 is sufficient to serve in a neuroprotective role, we cannot yet exclude a similar role for UCP4 and UCP5. Even though there is evidence that UCP2 may combine metabolic and uncoupling functions, most experimental systems studying UCP2 have focused on its role in ROS regulation, and so it is this aspect that will be discussed most in the following sections.

## 4. Transcriptional and Translational Regulation of the UCP2 Gene

The UCP2 gene extends over 8.4 kb and consists of eight exons and seven introns, a structure shared with the other members of the UCP family. While the intron/exon boundaries are conserved, there is considerable variation in intron size among the UCP genes, suggesting the evolution of different regulatory mechanisms. Following two 5′ untranslated exons, exons 3/4, 5/6 and 7/8 encode the three structural repeats of the protein. The structures of the UCP2 gene and protein have been reviewed in detail elsewhere [2], but some features of this genomic structure are important to point out because they provide useful information about the regulation of UCP2 expression and activity.

**Figure 3:**
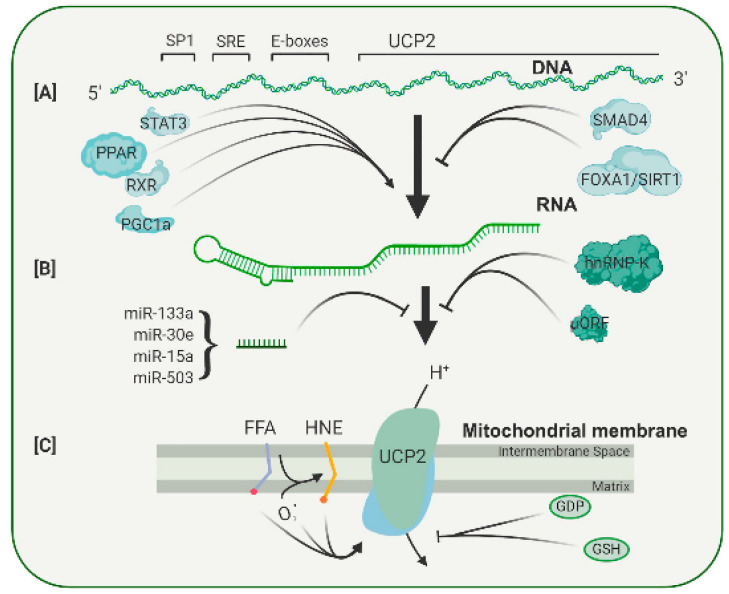
**Diagrammatic representation of the multiple levels of regulation of UCP2 expression and activity.** (**A**) UCP2 transcription is enhanced or repressed through binding of several transcription factors to sites in its promoter. (**B**) Translation of the UCP2 transcript is controlled by an upstream open reading frame (uORF), a number of microRNAs and RNS binding proteins, such as hnRNP-K. (**C**) The UCP2 protein can be activated by a number of compounds, including free fatty acids and lipid peroxides, and can be inhibited by the nucleotide GDP and by glutathione. A more complete discussion of the regulation of UCP2 expression can be found in Reference [2] from which this figure is adapted.

Because UCP2 is expressed in most tissues, it contains promoter elements that respond to common transcription factors, such as SP1 (Figure 3A). In addition, the UCP2 promoter contains a serum response element (SRE), which is likely to mediate the enhanced UCP2 transcription found after treatment with growth factors such as LIF and PEDF, both of which appear to act through a STAT3 signal pathway [33,34]. In the UCP2 promoter, two E-boxes are also present, but the transcription factors binding to these have not yet been identified. The E-boxes do, however, appear to be involved in the binding of PPAR family of transcription factors [35]. As shown in Figure 3, there is evidence that PPAR agonists increase expression of UCP2 in many tissues [36,37]. Treatment with the polyphenols resveratrol and quercetin caused an increase in levels of PPAR, and that, in turn, increased UCP2 RNA [38]. In addition to the PPAR factors themselves, a PPAR cofactor, PGC-1, can also regulate UCP2 transcription. Because PGC-1 and the PPAR factors are involved in the regulation of genes active in a wide range of metabolic pathways, particularly in mitochondria, it seems that the levels of UCP2 transcription are coupled to the general metabolic state of a cell. In addition to these metabolic conditions, UCP2 transcription can be affected by growth factors, signifying another integrative level of control (Figure 3). For example, TGF stimulates binding of SMAD4 to repressive elements in the UCP2 proximal promoter, and this decreases its transcription [39]. On the other hand, PEDF increases the levels of UCP2 RNA and restores resistance to oxidative stress in aged RPE cells [34]. The regulation of UCP2 transcription appears to be subtle, without dramatic increases or decreases, and there is growing evidence that the major control of UCP2 expression is through post-transcriptional mechanisms.

UCP2 is a relatively minor protein of mitochondria, and its expression varies considerably among tissues. The spleen has the highest level of UCP2, but even here, the amount has been estimated as 160-fold less than that of UCP1 in brown adipose tissue, constituting 0.01 to 0.1% of mitochondrial membrane protein in various tissues [40,41]. Other tissues have up to 10-fold lower amounts of UCP2 than the spleen. This is in contrast with the ANT proteins that were found to be 6% of mitochondrial protein [42]. The first indication of post-transcriptional control of UCP2 levels came from experiments in which the RNA and protein were measured in samples under different conditions. LPS treatment increased UCP2 protein levels by 12-fold without any measurable change in RNA levels [41]. Since then, at least three post-transcriptional regulatory mechanisms have been described (Figure 3B).

The first is a widely expressed RNA binding protein, hnRNP-K, which binds to sites in the 3′-UTR of UCP2 RNA. This binding is regulated by several factors, including insulin, angiopoietin and adiponectin, which can cross the blood–brain or blood–retina barrier and link UCP2 levels to the general metabolic status of the organism [43].

The second regulatory mechanism operates at the 5′ untranslated region of the UCP2 RNA. Within this region, a 36-amino-acid open reading frame has been identified [44]. This sequence has an inhibitory function, since mutations within the open reading frame lead to increased UCP2 protein expression. Activity of this uORF can be regulated by glutamine, a neurotransmitter precursor. Such regulation again suggests that UCP2 levels are regulated by the metabolic status of a cell [45,46].

The third potential regulatory mechanism is less well defined. There is evidence that a number of microRNAs can alter UCP2 RNA levels in many tissues (Figure 3B). Most studies show that stress or disease lowers the level of microRNA and leads to an increase in UCP2 protein [47]. Many of these studies are correlative, although in a few cases, specific microRNAs were shown to bind to the 3′-untranslated UTR of UCP2 RNA [48]. Some of these microRNAs are also found in the eye, but whether they play a role in UCP2 responses to retinal stress has yet to be determined.

One of the consequences of the complex post-transcriptional regulation of UCP2 is a rapid change in protein levels in response to modulations in the environment. This is primarily because the protein has a very short half-life. In several tissues, the half-life of UCP2 (and UCP3) has been measured as 1 h, considerably shorter than that of other inner mitochondrial membrane proteins, which have reported half-lives of between 4 and 17 days [49,50,51]. Isolated mitochondria do not show rapid UCP2 turnover, so it is thought that protein degradation is regulated by specific cytoplasmic factors [50]. Although these are not characterized, it is known that UCP2 enters the mitochondria via a series of specific chaperones and by a recognition sequence on UCP2 itself [52,53]. The rapid turnover kinetics of UCP2 and the multiple post-transcriptional mechanisms that link its expression to cell metabolism both argue for a key role of this protein in mitochondrial function and its potential to combat disease and injury.

## 5. Pharmacological Regulation of UCP Activity

In general, there is a correlation between UCP2 expression and function (with overexpression leading to increased uncoupling activity). UCP2 is, however, regulated by a number of allosteric modulators [2]. These include glutathione, mitochondrial matrix superoxide, GDP and fatty acids (Figure 3C).

It was found that induction of glutathionylation with diamines in thymocytes inactivated proton leakage in a UCP2-specific manner. The primary site of glutathionylation is thought to be cys^256^ based on studies with UCP3 and the conserved amino acid sequence [54]. ROS were able to reverse this glutathionylation, indicating that ROS and glutathione act together to regulate mitochondrial proton leak and ROS levels. This process is also linked to glucose metabolism, as elevated glucose levels decreases glutathionylation and the UCP2-mediated proton leak [55].

Regulation by GDP appears to be part of the network integrating ATP synthesis, mitochondrial membrane potential and ROS production [56]. When nucleotide triphosphate concentrations are reduced, GDP concentrations are usually increased. When GDP inhibits UCP2, it increases the mitochondrial membrane potential and ATP synthesis. GDP binds inside the UCP2 cavity, and this allosterically dislocates a fatty acid molecule from its binding site, which in turn inhibits UCP2 activity [20].

There is some debate about the role of fatty acids in regulating UCP2 activity. Many UCP2 models invoke a fatty acid molecule as the proton carrier that flips from side to side to give net proton flux [57]. Some have suggested that fatty acid binding to UCP2 serves as an allosteric effector that participates in the shift in substrate preference between glucose and fatty acids as substrate choice [29].

Of particular interest is the feedback regulation provided by the peroxidized fatty acid 4-hydroxynon-2-enal (4-HNE). 4-HNE is an aldehydic lipid peroxidation intermediate formed as a result of ROS interaction with unsaturated fatty acids and serves as a gauge of ROS production [58]. 4-HNE can activate the UCP2-mediated proton leak and thus decrease ROS production. 4-HNE is toxic, and higher concentrations can often damage cells by forming adducts with many proteins through their reactive aldehyde groups and through covalent interactions with aminophospholipids [59]. As described later, this prompted our successful search for 4-HNE mimetics to regulate UCP2 activity and provide protection for neurons from oxidative stresses.

## 6. UCPs and Neural Degeneration

Although acute and chronic neurological conditions may have different etiologies and symptoms and affect different brain regions, the end result of all of them is death of neurons. Oxidative stress has been identified as a major component of many of these conditions. In many such cases, degeneration occurs when the homeostatic mechanisms regulating ROS production and destruction are overwhelmed. Since a large component of the oxidative burden facing cells arises from ROS produced in mitochondria, this raises the interesting therapeutic possibility of enhancing these homeostatic mechanisms and providing cells with a greater tolerance of extrinsic oxidative stress. A strong case has been made for reducing mitochondrial stress and improving their function by using neuroprotective peptide factors, such as PEDF, and small molecules, such as CAPE [34,60]. There is now a growing body of literature to support the idea that UCPs, particularly UCP2, can modulate mitochondrial ROS generation and that they can provide significant protection in a variety of diseases.

Apart from the thermogenic action of UCP1, the most intensively studied uncoupling protein is UCP2. There is a body of evidence showing that UCP2 is involved in many degenerative conditions, either directly through its uncoupling function or indirectly through its overall action on mitochondrial homeostasis. Some of the first evidence for a role for UCP2 came in studies of atherosclerosis where UCP2 knockout mice showed much higher oxidative stress and larger atherosclerotic plaques than wild-type mice [61]. Conversely, mice overexpressing UCP2 were protected from the effects of global ischemia [62]. Similarly, in UCP2 overexpressing mice, brain damage was reduced compared with wild-type mice in experimentally induced stroke [63]. These same studies showed that UCP2 reduced cell death and caspase 3 activation induced by oxygen and glucose deprivation. Using a drug-induced model of grand mal seizures in mice, we were able to show that UCP2 overexpression reduced hippocampal neuron death [64]. In younger wild-type animals, protection against seizure-induced excitotoxic cell death was achieved by using dietary methods (high fat) to increase the basal levels of UCP2 [65].

A well-established model of neurodegeneration involving oxidative stress is the degeneration of substantia nigra neurons induced by the neurotoxin MPTP. The active form of this compound, MPP+, rapidly produces symptoms that mirror many features of Parkinson’s disease. In a mouse model of Parkinson’s disease, we found that transgenic mice overexpressing UCP2 retained significantly more nigral dopamine neurons and had higher striatal dopamine levels after MPTP treatment than wild type (36% cell loss vs. 62% loss in substantia nigra) [66]. Conversely, UCP2 knockout mice showed approximately a two-fold reduction in dopamine neurons and dopamine levels than wild type following MPTP treatment. These data on cell loss correlated with the levels of ROS measured in nigral neurons, with overexpressing transgenics showing the lowest and UCP2 null mice the highest levels. In this study, it was also noted that UCP2 knockout mice had reduced mitochondria numbers. This was confirmed in a more recent study that indicated increased mitophagy in UCP2 knockout animals [28]. These data argue that UCP2 activity plays an important role in maintaining mitochondrial numbers and quality. The studies on the mouse model of Parkinson’s disease confirmed our earlier findings in a primate model where we activated UCP2 by feeding the animals a UCP2 activator, Coenzyme Q_10_ (CoQ_10_) [67]. This treatment induced uncoupling, as defined by increased state 4 respiration. Following administration of MPTP, the CoQ10-treated animals showed significantly lower loss of dopamine cells in the substantia nigra than untreated animals (13 vs. 74%). Though less direct than the mouse studies, the work with primates suggests that UCP2 activators are strong therapeutic candidates to treat Parkinson’s disease and possibly other neurological conditions.

While most of the current data for the involvement of uncoupling proteins in neurodegenerative disease come from studies on UCP2, there is some evidence that UCP4 and UCP5 may play a role in Parkinson’s disease as well. Both UCP4 and UCP5 are expressed in the brain, though with different levels in different brain regions [68]. Overexpression of either UCP4 or UCP5 led to decreased mitochondrial membrane potential in neuronal cell lines and model organisms [69,70,71]. UCP4 protected cultured SH-SY5Y cells from MMP+ toxicity and PC12 cells from 3-NP toxicity [72,73]. Similarly, UCP5 protected against oxidative stress induced by MMP+ in SH-SY5Y cells [69]. There is still debate about whether UCP4 and UCP5 act as true uncoupling proteins or proteins whose transport mechanisms are recruited to provide neuroprotection [69]. Because UCP3 expression is confined to muscle, there are few studies associating it with disease. It appears that UCP3 is not involved in uncoupling but rather in the transport of a number of metabolic intermediates [74].

## 7. UCP2 and Retinal Degeneration

All retinal neurons and one of the retinal glia, the Müller cells, are derived from the retinal epithelium, an early outgrowth of the forebrain vesicle. The other layer of this outgrowth becomes the retinal pigment epithelium that forms part of the blood–retinal barrier and is critical in maintaining photoreceptors and other outer retinal cells. Other cell types found in the retina, including microglia, vascular elements and astrocytes, all migrate into the retina during development. While retinal neurons and Müller glia are derived from a common multipotential precursor, there subsequent developmental pathways yield mature cells with very different phenotypes and different susceptibilities to degenerative disease.

A key feature when considering the role of mitochondria and their responses in disease is that they are less able to withstand oxidative stress as they age. When human retinal pigment epithelial cells (RPE cells) from young (9–20 years), middle-aged (48–60 years) and advanced-age (62–76 years) donors were tested for susceptibility to H_2_O_2_ toxicity, they showed a sensitivity related to chronological age [75]. In addition, there was an age-related decrease in expression of UCP2 and superoxide dismutase 2. These molecular changes were in addition to clear differences in mitochondrial number, size, shape matrix density, cristae architecture and membrane integrity that were found with the older-aged samples. Such changes in aging mitochondria are undoubtedly a major contributor to the age-related diseases found in the retina and other parts of the nervous system.

Rod photoreceptors are the target of over 100 gene mutations leading to the inherited disease retinitis pigmentosa (RP) that affects approximately 1 in 4000 in the world. Cone photoreceptors are the target of age-related macular degeneration (AMD) that currently affects almost 200 million people worldwide. Diabetic retinopathy (DR), a major complication of diabetes, affects over 100 million people worldwide and is the leading cause of blindness among the working-age population. In addition to neuronal degeneration in the inner retina, DR results in serious disruptions of the retinal vasculature. Glaucoma is almost as prevalent with close to 80 million people worldwide suffering from this disease. The end result of glaucoma is the loss of retinal ganglion cells and optic nerve fibers. In the eye, the primary open angle glaucoma may be exacerbated from calcium-induced oxidative stress, which increases mitochondrial vulnerability and dysfunction in the trabecular meshwork, resulting in failure to control intraocular pressure [76]. Mitochondrial decay, bioenergetic deficits and weakened antioxidant defenses were also noted with increased aging in RPE cells [77]. The causes of many of these retinal degenerative diseases are not fully understood, but one thing in common is an increase in oxidative stress, and this is believed to be a major contributing factor to the degeneration.

While the majority of this review is characterizing oxidative stress as damaging for cells, we also need to consider that it is used as a normal signaling mechanism. Regulation of neuronal number is a complex event and during development often involves an initial overproduction of cells followed by loss of cells that have not made the appropriate connections. This process is often termed developmental or programed cell death. In mouse retina, there is an initial generation of 131,000 to 224,000 ganglion cells that becomes reduced to 45,000 to 76,000, depending on the mouse strain and methods used for counting [78,79]. When we examined the ganglion cell layers of wild-type, UCP2-overexpressing and UCP2 knockout mice, we found that overexpressors had 48% more cell bodies in the ganglion cell layer, and the UCP2 knockouts had 20% fewer [31]. This suggests that UCP2 play a role in programed cell death. TUNEL and cresyl violet staining of ganglion cell nuclei during the period of programed cell death revealed fewer degenerating cells in the transgenic animals. These data led us to suggest that programed cell death involves oxidative stress and/or mitochondrial signals and that these can be altered by increased UCP2.

### 7.1. Diabetic Retinopathy

Approximately 30% of diabetics are expected to develop diabetic retinopathy during their lives. Early, non-proliferative stages of the disease often progress into proliferative diabetic retinopathy in which there is abnormal growth of leaky blood vessels, edema and progressive vision loss. There is still debate as to whether diabetic retinopathy is a vascular disease, and there is increasing evidence that the vascular complications are a result of earlier neuronal loss and inflammation [80,81]. Diabetic hyperglycemia induces oxidative stress in the retina, which is a major cause of the microvascular component of diabetic retinopathy and is likely to affect the neurodegeneration and inflammatory components as well. Preventing mitochondrial ROS production blocks hyperglycemic damage in this tissue [82]. In cultured endothelial cells and pericytes, elevated glucose levels (23 mM) cause an increase in UCP2 transcripts, but higher glucose levels (above 30 mM) cause a reversion to control levels [83], suggesting that UCP2 exerts a homeostatic effect within a range of glucose concentrations but that this can be overwhelmed by higher glucose levels. A more direct link between UCP2 and vascular endothelial cells tolerance to elevated glucose was found when UCP2 knockdown resulted in increased apoptosis, and overexpression of UCP2 reduced caspase 3 activation and apoptosis [84,85]. A number of other responses of endothelial cells, pericytes and Muller glial cells, as well as a number of studies of antioxidants in cell culture, all indicate that reducing oxidative stress is beneficial at combating the broad effects of elevated glucose on retinal pathophysiology [86,87].

One of the most hopeful new therapeutic approaches to treat all the features of diabetic retinopathy is the finding that peptide mimetics of the neurotrophic factor PEDF applied as eyedrops decreases neuronal loss, inflammation and vascular abnormalities in rodent models [88]. Although PEDF acts through multiple receptors, one clear action is to increase UCP2 expression. In studies of RPE cells, it was found that susceptibility to oxidative stress increased with aging and that this correlated with decreased UCP2 transcripts [34]. PEDF blocks the stress-induced cell death, possibly by increasing UCP2 levels [89]. While the link between a factor that effectively treats diabetic retinopathy and the expression of UCP2 is currently only a strong correlation, it does argue for one avenue developing new therapeutics for this disease.

In an attempt to obtain more direct evidence for the involvement of UCP2 in diabetic retinopathy, several groups have carried out GWAS studies and have identified a significant linkage between several UCP2 polymorphisms and the diabetic retinopathy phenotype [90,91,92]. Interestingly, the association was stronger with the more severe, proliferative forms of the disease.

While UCP2 is clearly not the major cause of diabetic retinopathy, it seems to play an important role in the disease progression. Thus, enhancing UCP2 activity may provide strong therapeutic benefit.

### 7.2. Glaucoma

The group of progressive optic neuropathies termed glaucoma are all associated with retinal ganglion cell loss [2,93]. The most common forms of the disease affect adults over the age of 40. The most common risk factor for glaucoma is elevated intraocular pressure (IOP), and a number of drugs have been developed to lower IOP and slow the progression of glaucoma. Many forms of glaucoma, however, are not associated with high IOP, and even those that can be treated with IOP-lowering drugs still have slow neurodegeneration.

Tissues from glaucoma patients, and from animal models of glaucoma, have increased DNA oxidation, lipid peroxidation, protein carbonyl adducts and reduced mitochondrial function [94,95,96,97]. All of these findings support the idea that oxidative stress and damage is a key component of glaucoma.

To test whether UCP2 may be a useful target to reduce oxidative damage in glaucoma, we established a series of inducible transgenic mouse lines that would either overexpress or delete UCP2 selectively in retinal ganglion or glial cells [28,98]. We then generated animals with elevated IOP using polystyrene bead injections into the anterior chamber of the eye to block aqueous outflow. Wild-type mice treated in this way show a gradual loss of retinal ganglion cells over time. Overexpression of UCP2 in retinal ganglion cells significantly reduced cell death in the high IOP animals. Overexpression of UCP2 in glial cells, however, had no effect, suggesting that the actions of UCP2 are predominantly cell autonomous.

One surprising result from these studies was that deletion of UCP2 in either ganglion cells or glia did not increase cell death in the high IOP animals. Further studies indicated an increase in ROS in glial cells, but the major effect of UCP2 deletion was an increase in mitophagy. By reducing the numbers of mitochondria, particularly damaged mitochondria, ganglion cells were better able to survive.

### 7.3. Age-Related Macular Degeneration (AMD)

With over 200 million people suffering from AMD in the world, it ranks as one of the most prevalent neurodegenerative diseases. The characteristic effect of AMD is central blindness due to loss of cone photoreceptors, primarily in the central, or macular, region of the retina. There are still debates about the relative importance of RPE cells and cone photoreceptors as the initiating cells for AMD, but no debate about the correlation with aging. The progression of AMD involves changes in RPE cells, the accumulation of extracellular deposits (drusen) and the death of cone photoreceptors [99]. This “dry” form of AMD can develop into geographic atrophy where significant portions of the macula are affected. A significant number of AMD patients later develop the “wet” form of the disease in which choroidal neovascularization invades the retina and causes rapid loss of photoreceptors and vision. While the initial causes of AMD are still obscure, GWAS studies have identified several loci associated with the dry and wet forms of AMD, respectively [100,101]. Other risk factors have subsequently been described, many of them linked to the complement pathway [102,103]. Other risk factors include mitochondrial DNA haplogroups. We have shown that the J mtDNA haplogroup alters the RPE transcriptome in transmitochondrial cybrids, such that the cells become increasingly susceptible to oxidative stress compared to H haplogroup cybrids, possibly due to their downregulation of many genes involved in mitochondrial complex 1 and V function [104]. While current anti-VEGF therapeutics are effective at blocking the rapid loss of vision caused by blood vessel growth in the wet form of AMD, they are ineffective for approximately 30% of patients, lose effectiveness over time and do not prevent the development of geographic atrophy [105].

There is substantial evidence for oxidative stress in AMD, but its place in the disease initiation and progression is still debated [106]. The oxidative insults affecting cone photoreceptors and RPE cells are both extrinsic and intrinsic. Extrinsic oxidative agents include sunlight, smoking and a number of dietary compounds. RPE cells in particular are subjected to stress both from light-induced reactions and from their proximity to the oxygen-rich choroidal blood supply. The effects of many of these insults can be lessened by anti-oxidant compounds and stimulation of cytoplasmic anti-oxidant enzymes. Intrinsic oxidative stress is generated in mitochondria because of the very high metabolic rate of photoreceptors and RPE cells. This high flux through the oxidative phosphorylation chain produces levels of ROS that need to be neutralized. Uncoupling proteins can achieve this but at the risk of decreasing ATP production. As discussed earlier, the correlation of mitochondrial driving force and ROS is not linear, and a small decrease in driving force can result in a large decrease in ROS. Careful titration of UCP2 activity by large arrays of regulatory compounds can achieve the optimal balance between energy production and ROS production.

## 8. UCP2 as a Target to Combat Retinal Degeneration

In the sections above, we described how UCPs, particularly UCP2, play a key role in regulating oxidative stress and that altering UCP2 activity can provide significant protection. Most of these studies, however, used genetic manipulation to alter UCP2 levels and activity. This is clearly not applicable to a patient population. Nevertheless, we now have strong data indicating that UCP2 activity can be modulated by small molecules.

Early studies identified genipin as an inhibitor of UCPs. Genipin is a natural cross-linker isolated from *Gardenia jasminoides Ellis* and is found in many traditional Chinese medicines. Genipin is able to freely cross the cell membrane and inhibits UCP2-mediated “proton leakage” at the mitochondrial level. It can increase mitochondrial membrane potential, increase ATP and ROS levels and is used as an ideal inhibitor for studying UCP2 function in vivo [107]. In particular, it has proven very useful in delineating uncoupling and other mechanisms that are dependent on UCPs.

**Figure 4:**
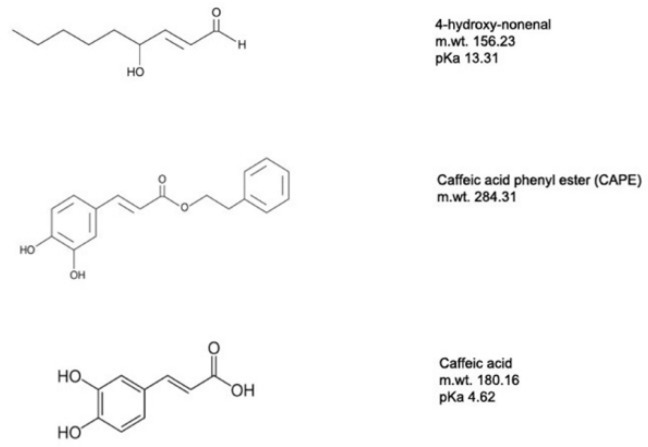
The molecular structures of 4-Hydroxynon-2-enal (4-HNE), caffeic acid phenyl ester and caffeic acid. Adapted from Reference [60].

**Figure 5:**
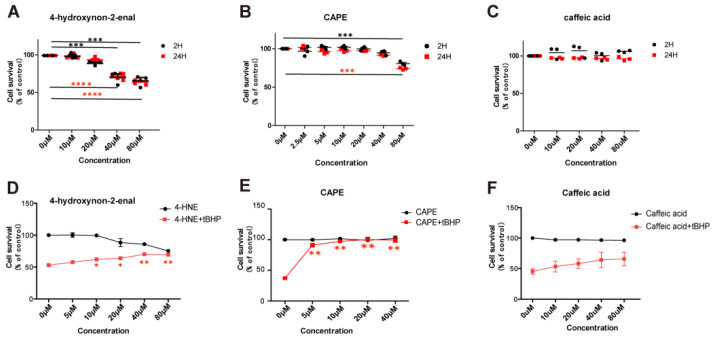
**CAPE is nontoxic and can prevent peroxide-induced cell death.** (**A**–**C**). Cell viability was measured by an LDH assay after pre-incubation with different concentration of (**A**) 4HNE; (**B**) CAPE; (**C**) caffeic acid for 2 h (black symbols) or 24 h (red symbols). Data are expressed as individual points as well as mean ± SD. The 24 h group statistical differences are shown in red (n = 3, * *p* < 0.05, ** *p* < 0.01, *** *p* < 0.001, **** *p* < 0.0001); 2 h group statistical differences are shown in black (n = 3, * *p* < 0.05, ** *p* < 0.01, *** *p* < 0.001. (**D**–**F**). ARPE19 cells were treated for 12 h with different concentration of compounds without (black curve) or with (red curve) t- BHP (200 μM). (**D**) 4HNE; (**E**) CAPE; (**F**) caffeic acid. The decrease in cell survival induced by t-BHP was completely blocked by CAPE. 4-HNE, and caffeic acid gave smaller improvements in cell survival and required higher concentrations. Data points represent mean ± SD, n = 3. Error bars are only shown when they are larger than the data point symbols. Adapted from Reference [60].

**Figure 6:**
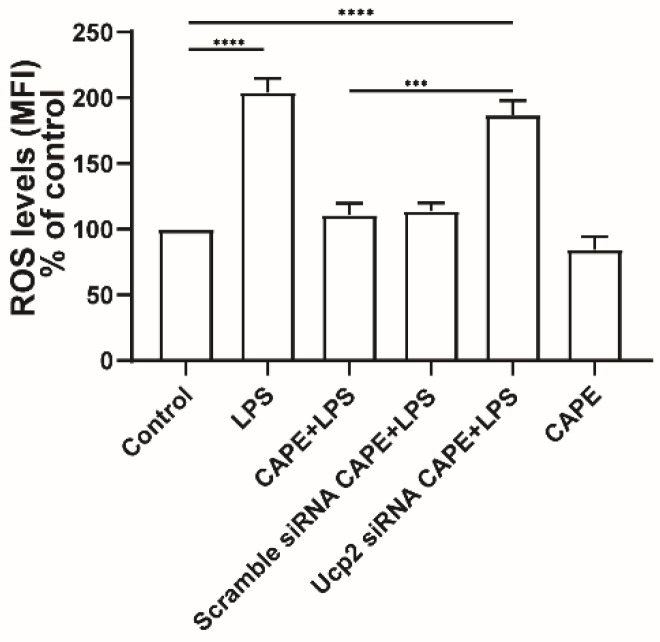
**Effect of UCP2 knockdown on ROS production in ARPE19 cells.** ROS levels were determined by Mitosox red fluorescence. The mean fluorescence intensities (MFI) were analyzed by flow cytometry software (FlowJo). (n = 3 *** *p* < 0.001, **** *p* < 0.0001). Adapted from Reference [60].

**Figure 7:**
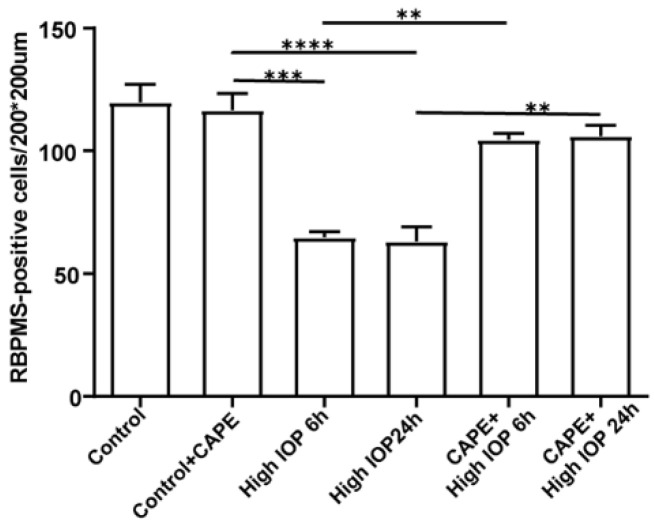
**CAPE enhances retinal ganglion cell survival in the retina 6 h or 24 h after transient ischemia/reperfusion.** The number of RBPMS-positive cells were counted from flat mounts of retina. The histogram shows the mean ± SD, n = 4; ** *p* < 0.01 *** *p* < 0.001, **** *p* < 0.0001. Adapted from Reference [60].

Recently, we took a different approach and looked for mimetics that have some structural similarity to the natural UCP2 activator 4-HNE but with less toxicity [60]. Among the compounds tested were caffeic acid and its phenyl ester, CAPE (Figure 4). Both compounds were less toxic than 4-HNE and capable of reducing ROS production induced by LPS treatment of cell cultures, though CAPE was much more efficient at this as well as at reducing cell death induced by either LPS or t-BHP (Figure 5). It is likely that CAPE was more efficient because, as an ester, it is more cell permeable than the free acid, caffeic acid. In those studies, the action of CAPE was dependent on UCP2, since both inhibition by genipin and siRNA knockdown blocked CAPE’s activity (Figure 6). We also tested the action of CAPE in an acute animal model of glaucoma, retinal ganglion cell death induced by ischemia/reperfusion. Daily intraperitoneal injections of 10 mg of CAPE substantially reduced the ganglion cell death resulting from 60 min of ischemia induced by raising the intraocular pressure to 50 mmHg by connection of the anterior chamber of the eye to an elevated saline reservoir (Figure 7).

## 9. Conclusions and Future Directions

In the above sections, we discussed the role of UCP2 and mitochondria in several neurodegenerative diseases. At times, it may appear that the terms uncoupling protein and mitochondria are used interchangeably. We argue that this is because uncoupling proteins regulate many of the actions of mitochondria. Changes in ROS production, mitophagy and mitochondrial number, as well as in substrate utilization, have all been linked to uncoupling proteins, primarily UCP2. Although the exact mechanism(s) of action of UCP2 have yet to be fully defined, the manipulation of the expression or activity of UCP2 leads to such strong effects on neurodegenerative disease that this protein is clearly a high priority as a drug target. Given the many ways in which UCP2 levels and activity can be manipulated, it is likely that therapeutics interacting with this protein will soon be produced. The eye, and particularly the retina, is an ideal system to test such therapeutics. There are a series of retinal degenerations that are major health problems, and good animal models of many of them are available. Even more importantly, there are excellent non-invasive ways of monitoring retinal function that allow longitudinal studies of the same animals, as well as monitoring efficacy in human trials. Because the eye is a uniquely isolated organ, delivery of drugs to the retina is possible with only minimal, or no, side effects on other organs. We propose that the most promising lines of research into UCP2 therapeutics lie in the development of small molecule activators, such as CAPE or related compounds. Topical application of such compounds may provide a simple approach to tackle a number of currently intractable degenerative eye diseases.
